# Effects of commercial direct-fed microbials and zinc-methionine supplements on growth performance in preweaning Holstein calves under commercial rearing conditions

**DOI:** 10.1016/j.vas.2026.100845

**Published:** 2026-08-31

**Authors:** Ahmadreza Alipour, Mahdi Ganjkhanlou, Abolfazl Zali, Mahdi Dehghan-Banadaki, Hossein Esmaeili, Mohammad Ramin

**Affiliations:** a Department of Animal Science, Faculty of Agriculture, College of Agriculture and Natural Resources, University of Tehran, Karaj, 7787131587, Iran; b Department of Microbiology and Immunology, Faculty of Veterinary Medicine, University of Tehran, Tehran, 141556619, Iran; c Department of Applied Animal Science and Welfare, Faculty of Veterinary Medicine and Animal Science, Swedish University of Agricultural Sciences, Umeå, 90183, Sweden

**Keywords:** Calf, Blood parameters, Probiotics, Trace minerals

## Abstract

This study was conducted to evaluate the effects of direct-fed microbials (DFM) and zinc-methionine (ZM) as well as their potential interaction and dose response of ZM as nutritional strategies to improve growth performance, blood metabolites, rumen fermentation characteristics, and apparent nutrient digestibility in preweaning Holstein calves. Seventy-two newborn Holstein calves (4 d of age, BW = 38.6 ± 6.27 kg) were assigned to a 2 × 3 factorial arrangement of treatments with DFM at 2 levels (0 or 5 g/calf/d) and ZM at 3 levels (0, 422, and 632 mg/calf/d). Main effects of DFM and ZM increased body weight, final body weight and dry matter intake. As ZM dose increased, body weight increased both linearly and quadratically, while final body weight increased quadratically and dry matter intake increased linearly. Main effect of DFM increased average daily gain. Withers height increased with main effects of DFM and ZM, whereas heart girth increased with main effects of DFM and ZM and also DFM × ZM interaction. Hip width increased by ZM main effect. Zinc-methionine level increased heart girth both linearly and quadratically but increased hip width quadratically and withers height linearly. In plasma, DFM main effect increased glucose, total protein, and cholesterol, whereas ZM main effect increased albumin. Plasma albumin increased linearly in response to ZM level. According to our results, DFM and ZM can be considered as effective additives to improve growth performance in preweaning dairy calves. No consistent evidence was found that combined DFM and zinc supplementation might enhance calf performance.

## Highlights

• Direct-fed microbials and zinc-methionine improved calf growth.
• Supplementation increased starter intake and final body weight.
• Both additives supported skeletal development before weaning.
• Growth gains were improved by intake.
• Combined DFM and zinc-methionine showed limited synergy.

## 1 Introduction

Dairy calves’ growth and development during the preweaning stage influence their future productivity (Drackley et al., 2026). Therefore, effective nutritional strategies which ensure optimal average daily gain (ADG) and weaning weight in preweaning calves, improve their subsequent productivity (Palczynski et al., 2020). Thus, preweaning nutrition can be viewed as a strategic investment that influences future productivity (Palczynski et al., 2020).

Direct-fed microbials (DFM) have been suggested as an effective method to optimize performance in preweaning calves (Cangiano et al., 2020). Direct-fed microbials may optimize the intestinal microbiome by competing with pathogens for nutrients or binding sites, as well as making the environment unfavorable for their growth by producing lactic acid and lowering pH in small intestine (Callaway & Ricke, 2012; Lee et al., 2025). In multispecies DFM, compatible strains may exert complementary functions and synergistic interactions by occupying different binding sites, facilitating each other’s colonization by optimizing the intestinal environment, producing a broader spectrum of antimicrobial metabolites, and collectively enhancing competitive exclusion of pathogens and modulation of host immune responses (Lambo et al., 2021; Timmerman et al., 2004). These complementary activities may improve the stability and functionality of the intestinal microbiota more effectively. Several meta-analyses have reported that DFM supplementation may improve calf performance, including increases in dry matter intake (DMI) and ADG (Alawneh et al., 2020; Branco-Lopes et al., 2025; Wang et al., 2023).

Zinc has a stimulating effect on DMI by modifying the appetite hormones such as leptin, cholecystokinin, and ghrelin (Suttle, 2022). Zinc also affects signaling pathways which organize cell response to insulin-like growth factor as well as regulating genes involved in redox reactions, growth and energy utilization (Suttle, 2022). Zinc-methionine (ZM) is an organic trace mineral characterized by enhanced bioavailability compared with inorganic zinc sources, due to its chelation with methionine, which facilitates intestinal absorption and systemic utilization of zinc (Cai et al., 2025). It has been shown that supplementation of 80 or 120 mg/d zinc as organic sources has improved ADG, DMI and feed efficiency linearly in preweaning calves (Liu et al., 2023b; Wo et al., 2022). Some other studies have reported that supplying 80 mg/d of zinc in chelated form including ZM has increased ADG and FE in dairy calves (Chang et al., 2020; Liu et al., 2023a).

Zinc may facilitate DFM colonization via effects on mucin gene expression, whereas DFM may enhance zinc absorption through luminal acidification and short-chain fatty acid production that supports intestinal development (Dudík et al., 2020; Varvara & Vodnar, 2024). However, evidence from controlled studies evaluating combined supplementation in ruminants remains scarce. For example, one study found that supplementation with a mixture of trace minerals and DFM improved feed efficiency in Awassi lambs (Alhidary et al., 2016). But to our knowledge, no study has investigated the dual effects of DFM and ZM as growth promoters in preweaning dairy calves. Moreover, most previous studies have evaluated organic zinc supplementation only during the first 2 to 4 weeks of life (Hashemi, 2025). Also, most DFM studies in preweaning calves have not evaluated plasma metabolic indices alongside performance, digestive and rumen parameters (Branco-Lopes et al., 2023).

Accordingly, the objective of this study was to determine the main effects of commercial DFM and ZM, their potential interaction and dose response of ZM on growth performance, blood metabolites, rumen fermentation characteristics, and apparent nutrient digestibility in preweaning calves under practical rearing conditions.

## 2 Materials and methods

The procedures were carried out from May to September 2025 at Abooei commercial Dairy Farm, Mehriz, Yazd, Iran. The present study was authorized by the Animal Ethics Committee of College of Agriculture and Natural Resources, University of Tehran, with the authorization number AEC-202,504-1011.

### 2.1 Experimental design

Seventy-two newborn Holstein calves (36 males and 36 females; birth weight = 38.6 ± 6.27 kg) were randomly assigned to a completely randomized 2 × 3 factorial arrangement of treatments. Treatments consisted of 2 levels of DFM supplementation: no DFM or 5 g DFM/calf daily (providing 3 × 10^9^ CFU bacterial strains/calf/d and 5 × 10^8^ CFU yeast/calf/d). Zinc-methionine was supplemented at 3 levels: 0, 422 (providing 80 mg Zn/calf/d), or 632 mg/calf/d (providing 120 mg Zn/calf/d). The six treatment groups were then (1) No supplementation (DFM- ZM-), (2) Direct-fed microbials supplementation without zinc (DFM+ ZM-), (3) Low zinc supplementation without DFM (DFM- Low ZM), (4) High zinc supplementation without DFM (DFM- High ZM), (5) Low zinc supplementation with DFM (DFM+ Low ZM) and (6) High zinc supplementation with DFM (DFM+ High ZM). The DFM dose (5 g/d) used in this study was based on the manufacturer’s recommendation. The dose of zinc (80 and 120 mg/calf/d) was applied according to previous experiments (Chang et al., 2020; Liu et al., 2023b). The calves received supplements from d 4 until d 73 (weaning), for 70 d. The DFM and ZM supplements were mixed with whole milk and fed to the calves once daily during the morning feeding.

### 2.2 Direct-fed microbials and zinc-methionine supplements

The DFM used in the present study was a commercial multi-strain product (GUTROCARE Calf, Bioluence, Tehran, Iran) containing 6 × 10^11^ CFU/kg of each bacterial strain (*Bifidobacterium bifidum, Enterococcus faecium, Streptococcus thermophilus, Lactiplantibacillus plantarum, Lactobacillus delbrueckii* subsp. *bulgaricus, Lactobacillus acidophilus, Lacticaseibacillus rhamnosus, and Lacticaseibacillus casei*) and 1 × 10^11^ CFU/kg of *Saccharomyces cerevisiae*. The ZM supplement was also a commercial product (DeMet Zinc190, DeBon, Hengyang, China) that contained 190 g/kg zinc chelated with 420 g/kg methionine.

### 2.3 Calf management

The procedures were carried out at Abooei Dairy Farm, Mehriz, Yazd, Iran. Calves were housed individually immediately after birth in individual pens (1.67 m × 1.14 m × 1.08 m). The pens were made of enclosed concrete walls and bedded with sand. The barn was equipped with a custom-made cooling system consisted of three high-capacity (140 × 140) industrial ventilation fans (EIG industrial group) with two on the north side and one centrally located, and a wetted porous brick inlet wall on the south side. Continuous water flow over the porous blocks provided cooled inlet air that was distributed throughout the barn, maintaining an average ambient temperature of approximately 26 °C during the study. Ambient temperature was monitored using digital thermometers (HTC-2, Sinometer).

Calves received 6 L of colostrum (≥ 22% Brix) via bottle within the first 12 h of life, and after that 6 L of transition milk on d two and three at 08:00 and 17:00. The calves were fed whole milk at 08:00 and 17:00 using a bucket. From d 1 to d 62 of the trial (4 d of age to d 65), calves were fed 8 L/d of whole milk. From d 63 to d 70 of the experiment (66 d of age to 73), the milk allowance was reduced by 1 L/d each d. A mash starter was offered ad libitum to calves from the beginning of the trial. Based on a comprehensive review, the best time to offer forage to calves has been found to be about two weeks after birth (Xiao et al., 2020). Accordingly, from d 12 of the experiment (15 d of age), alfalfa hay with particle size of 2 to 3 cm was added to the starter at 100 g/kg of the starter. The starter was formulated according to NASEM recommendations (NASEM, 2021). Calves had free access to water throughout the trial. The starter ingredients and nutrient composition of starter and whole milk are shown in Table 1, Table 2, Table 3. Calves were monitored daily for general health throughout the trial using the Wisconsin Calf Health Scoring Criteria (McGuirk, 2008), including evaluation of rectal temperature, cough, nasal and ocular discharge, ear position, and fecal consistency. No calves died or were removed from the study.

**Table 1:** Ingredients of starter diet fed to the calves.

Item 1^,^^2^	*g*/kg (DM basis)
Corn grain	500
Soybean meal	340
Barley grain	50.0
Wheat bran	40.0
Soybeans, whole roasted	30.0
Sodium bicarbonate	10.0
Calcium carbonate	8.00
Di-calcium phosphate	5.00
Vitamin Premix	4.00
Bentonite	4.00
Trace Mineral Premix	3.00
Salt	2.00
Magnesium oxide	2.00
Toxin Binder	2.00

1 Each kilogram of mineral premix contains: Zinc 13,000 mg; cobalt 80 mg; selenium 100 mg; manganese 12,000 mg; copper 500 mg; iodine 120 mg per kg.

2 Each kilogram of vitamin premix contains: vitamin A 1200,000 IU; vitamin D3 250,000 IU; vitamin E 12,000 IU per kg.

**Table 2:** Nutrient values of the solid diet fed to the calves (DM basis^1^).

2Item	ME (Mcal/kg)	CP (%)	EE (%)	NDF (%)	Ash (%)	Calcium (%)	Phosphorus (%)	Zinc (mg/kg)
Starter	3.04	21.5	4.69	14.6	7.00	0.680	0.410	67.1
Alfalfa hay	1.98	14.8	1.21	46.6	9.97	1.36	0.280	24.3
Starter with 10% Alfalfa hay	2.92	20.9	4.37	17.5	7.20	0.750	0.390	63.5

DM = dry matter.

1 Starter DM = 89.2%; Alfalfa hay DM = 87.6%; Starter with 10% alfalfa hay DM = 89.1%.

2 ME = metabolizable energy; CP = crude protein; EE = ether extract; NDF = neutral detergent fiber.

**Table 3:** Nutrient composition of liquid diet (whole milk) fed to the calves.

Chemicals	%
Total solids	12.5
Fat	4.12
Solid not fat	8.37
Lactose	4.67
Protein	3.04

### 2.4 Intake and growth measurements

The body weight (BW), withers height (WH), hip width (HW) and heart girth (HG) of calves were measured at 07:30 before morning milk was fed on d 1, 14, 28, 42, 56 and 70 of the experiment. Body weight was measured using a digital livestock scale (WLC+P, Etemad industrial group). Withers height was measured using a measuring stick, whereas HG and HW were measured using a flexible measuring tape, following standard procedures for body measurements in dairy calves (Heinrichs et al., 1992). Average daily gain was calculated for each calf for consecutive measurement periods (d 1–14, 14–28, 28–42, 42–56, and 56–70) as the change in trait divided by the number of d in the corresponding periods (14). Starter intake was measured daily as the difference between the amount offered and refusals. Average daily starter intake (ADSI) was calculated as the change in intake divided by number of d in a week. Daily DMI was calculated from milk and SI based on their respective DM concentrations. Feed conversion ratio (FCR) was then determined by dividing DMI by ADG.

### 2.5 Sampling

Fecal grab samples were collected twice daily from rectum (morning and afternoon) during the last 3 d of the trial (d 68 to 70) and stored at −20 °C. Also, samples of starter and alfalfa hay were taken once a week and stored at −20 °C. Milk samples were also collected weekly from the milk offered to calves. At the end of the experiment (d 70), rumen fluid from the calves was collected by a custom-made stomach tube (approximately 150 cm long and 1.1 cm internal diameter) at 4 h after morning milk feeding on the last d of the study. The collected rumen fluid at first attempt was discarded (50 mL) to reduce saliva contamination. The final sample (50 mL) was filtered using 3 layers of cheesecloth. Then, 10 mL of rumen fluid was immediately mixed with 0.2 mL of 50% (v/v) sulfuric acid to halt further fermentation, enabling accurate determination of volatile fatty acids (VFA) and ammonia nitrogen (NH_3__—_N). Blood samples were taken from the vena jugularis of calves using heparin vacutainer tubes before the first meal at 07:30 on d 1, 35, and 70 of the trial. The tubes were kept on ice after collection and transported to the on-farm laboratory within less than an hour, then plasma samples were collected by centrifugation at 3000 *g* for 15 min (Z 200 A, Maschinenfabrik Berthold Hermle AG), and kept at −20 °C.

### 2.6 Diet nutrient composition and digestibility measurements

Metabolizable energy (ME), calcium, phosphorus, and zinc in the diet were estimated using the NASEM-Dairy-8 software (NASEM, 2021). The supplemental zinc doses (80 and 120 mg/calf/d) were considered the experimental variable of interest in this study, rather than the estimated total dietary zinc concentration. Milk samples were analyzed for total solids, fat, solids not fat, lactose, and protein using a milk analyzer (Lactoscan SP60, Milkotronic Ltd.). Fecal samples were dried in a forced-air oven at 65 °C for 48 h (Model Plus; GALLEKAMP). Dried samples of faeces and feed were then ground (FDR 90 L/4P, Dietz-Motoren GmbH & Co. KG) to pass a 1-mm screen. Dry matter of the feed and fecal samples were determined by oven-drying at 105 °C for 24 h (Plus oven, GALLEKAMP) according to AOAC method 930.15. Organic matter was calculated as the difference between DM and ash following complete combustion at 550 °C for 6 h based on AOAC method 942.05 using a muffle furnace (MR 170, Heraeus). Total nitrogen in feed and feces was measured by acid digestion followed by distillation and titration using the Kjeldahl procedure (KJELTEC AUTO 1030 Analyzer, Foss A/S), and CP was calculated as N × 6.25 following the AOAC method 990.03 (AOAC, 2023). Ether extract was quantified using a Soxhlet system (SoxTEC System HT 1043, Foss A/S) with petroleum ether as the solvent as stated by AOAC method 920.39. Neutral detergent fiber was analyzed according to the method presented by Van Soest et al. (1991). Apparent total-tract digestibility of DM, OM, CP, EE and NDF in calves was estimated using acid-insoluble ash as an internal marker, as described by Van Keulen and Young (1977).

### 2.7 Rumen fermentation parameters measurements

Rumen fluid pH was recorded immediately after collection with a portable pH meter (PP775, Zag chemie Yaran). For NH_3__—_N determination, rumen fluid samples were centrifuged at 3000 g for 15 min (Z 200 A, Maschinenfabrik Berthold Hermle AG). Subsequently, 40 µL of the supernatant were combined with 40 µL distilled water, 2.5 mL phenol reagent, and 2 mL alkaline hypochlorite reagent, and the reaction mixture was incubated in a water bath at 37 °C for 10 min. Ammonia nitrogen concentration was then measured spectrophotometrically (UV-2100, Shimadzu corporation) at 550 nm (Smith & Murphy, 1993). Volatile fatty acids (acetate, propionate, butyrate, isobutyrate, valerate, and isovalerate) were quantified by gas chromatography (GC-PU4410, Philips) equipped with a flame ionization detector using a packed column (2 m × 4.6 mm i.d., 10% PEG) with nitrogen as the carrier gas. The injector and detector temperatures were set at 200 °C (Ottenstein & Bartley, 1971).

### 2.8 Plasma biomarkers measurements

Plasma metabolites including glucose, β-hydroxybutyrate (BHB), total protein (TP), albumin, urea, creatinine, triglycerides and cholesterol were analyzed at diagnostic laboratory of Drug Applied Research Center, Tabriz University of Medical Sciences, Tabriz, Iran. Glucose and cholesterol were determined using Riton reagents (GL: 041–407; Chol: 027–407). Total protein, albumin, and urea were measured using Biorex-Fars kits (TP: 173–01–048/7; albumin: 222–01–053/4; urea: 124–01–041/6). Creatinine and triglyceride were analyzed using Delta kits (Creatinine: 1710,724; triglyceride: 1921,325). BHB was quantified using Randox kit (373RB).

### 2.9 Statistical analysis

All tests and data analyses were done using SAS (version 9.4). Normality of residuals was evaluated using the Shapiro-Wilk test and homogeneity of variance was assessed by Levene’s test.

Repeated measures outcomes including BW, WH, HW, HG, ADG, DMI, ADSI, FCR, glucose, BHB, TP, albumin, urea, creatinine, triglyceride and cholesterol were analyzed using PROC MIXED (Littell et al., 1998), under a completely randomized factorial arrangement (DFM: 2 levels; ZM: 3 levels) The model was:

$$Y_{\text{ijkl}}=\mu +\text{DF}M_{i}+ZM_{j}+T_{k}+{\left(\text{DFM}\times \text{ZM}\right)}_{\text{ij}}+{\left(\text{DFM}\times T\right)}_{\text{ik}}+{\left(\text{ZM}\times T\right)}_{\text{jk}}+{\left(\text{DFM}\times \text{ZM}\times T\right)}_{\text{ijk}}+\text{COV}+C_{l}+e_{\text{ijkl}}$$

Where, Y_ijkl_​ is the dependent variable; μ is the overall mean; DFM_i_​ is the fixed effect of DFM; ZM_j_ is the fixed effect of ZM; T_k_​​ is the fixed effect of sampling or measurement time; interaction terms represent the corresponding fixed interactions; COV is initial measurements as covariate (when appropriate); C_l_ is the random effect of calf; and e_ijkl_ is the residual error. Individual calf was considered as the experimental unit and subject for repeated observations. Sampling or measurement time was specified as the repeated factor. Since repeated measurements were obtained from the same calves at equally spaced time points and the data set was complete and balanced, a compound symmetry covariance structure was used to account correlations over time (Armstrong, 2017; Littell et al., 1998). Unrepeated variables, including FBW, apparent nutrients digestibility (DM, OM, EE, CP, and NDF) and ruminal parameters (pH, NH_3__—_N, total VFA, acetate, propionate, butyrate, isobutyrate, valerate and isovalerate), were analyzed using PROC GLM (SAS, 2023). The model was:

$$Y_{\text{ijk}}=\mu +\text{DF}M_{i}+ZM_{j}+{\left(\text{DFM}\times \text{ZM}\right)}_{\text{ij}}+e_{\text{ijk}}$$

Where, Y_ijk_ is the dependent variable; μ is the overall mean; DFM_i_ is the fixed effect of DFM; ZM_j_ is the fixed effect of ZM; interaction term represents the corresponding fixed interactions; and e_ijk_ is the residual error.

Linear and quadratic orthogonal contrast coefficients for the ZM levels were derived in SAS using PROC IML from the actual dose spacing (0, 422, and 632 mg ZM/calf/day), and the resulting coefficients were then tested using the LSMESTIMATE statement in SAS. Statistical significance was declared at *P ≤* 0.05, and trends were discussed at 0.05 *< P ≤* 0.10. Mean values presented in this study are least square means obtained by LSMEANS statement in SAS. Inclusion of sex did not affect treatment estimates so it was removed in the final model.

## 3 Results

### 3.1 Growth performance

**Fig. 1:**
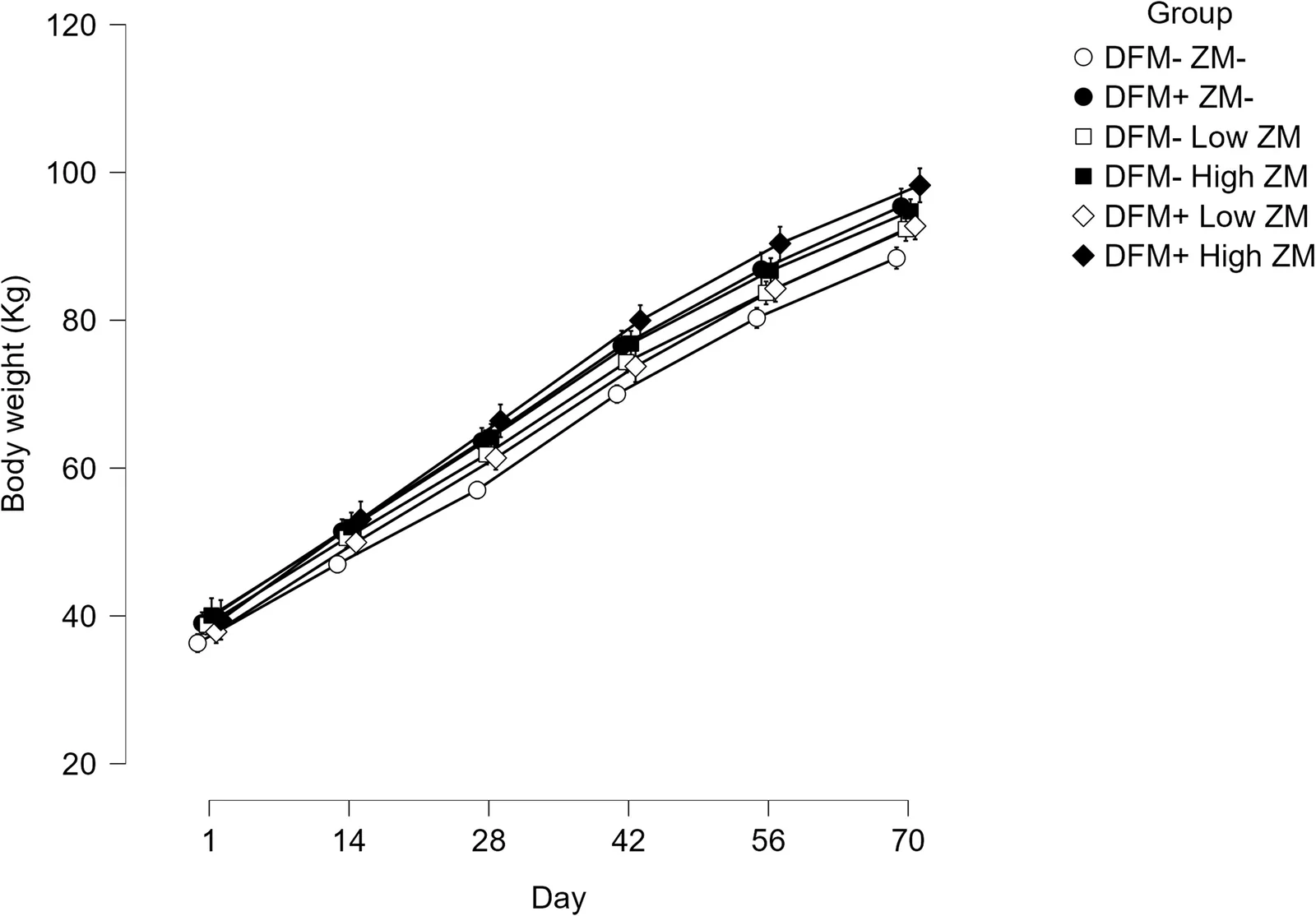
Body weight (BW) in calves receiving whole milk with or without supplementation of direct-fed microbials (DFM) and 2 levels of zinc-methionine (ZM) measured on d 1, 14, 28, 42, 56, and 70 of the experiment. Experimental groups were: (1) No supplementation (DFM- ZM-), (2) Direct-fed microbials supplementation (5 g/calf/d; providing 3 × 10^9^ CFU bacterial strain/calf/d and 5 × 10^8^ CFU yeast/calf/d) without ZM supplementation (DFM+ ZM-), (3) Low ZM supplementation (422 mg/calf/d; providing 80 mg zinc/calf/d) without DFM supplementation (DFM- Low ZM), (4) High ZM supplementation (632 mg/calf/d; providing 120 mg zinc/calf/d) without DFM supplementation (DFM- High ZM), (5) Direct-fed microbials supplementation with low ZM supplementation and (6) Direct-fed microbials supplementation with high ZM supplementation. The error bars represent the standard errors.

**Fig. 2:**
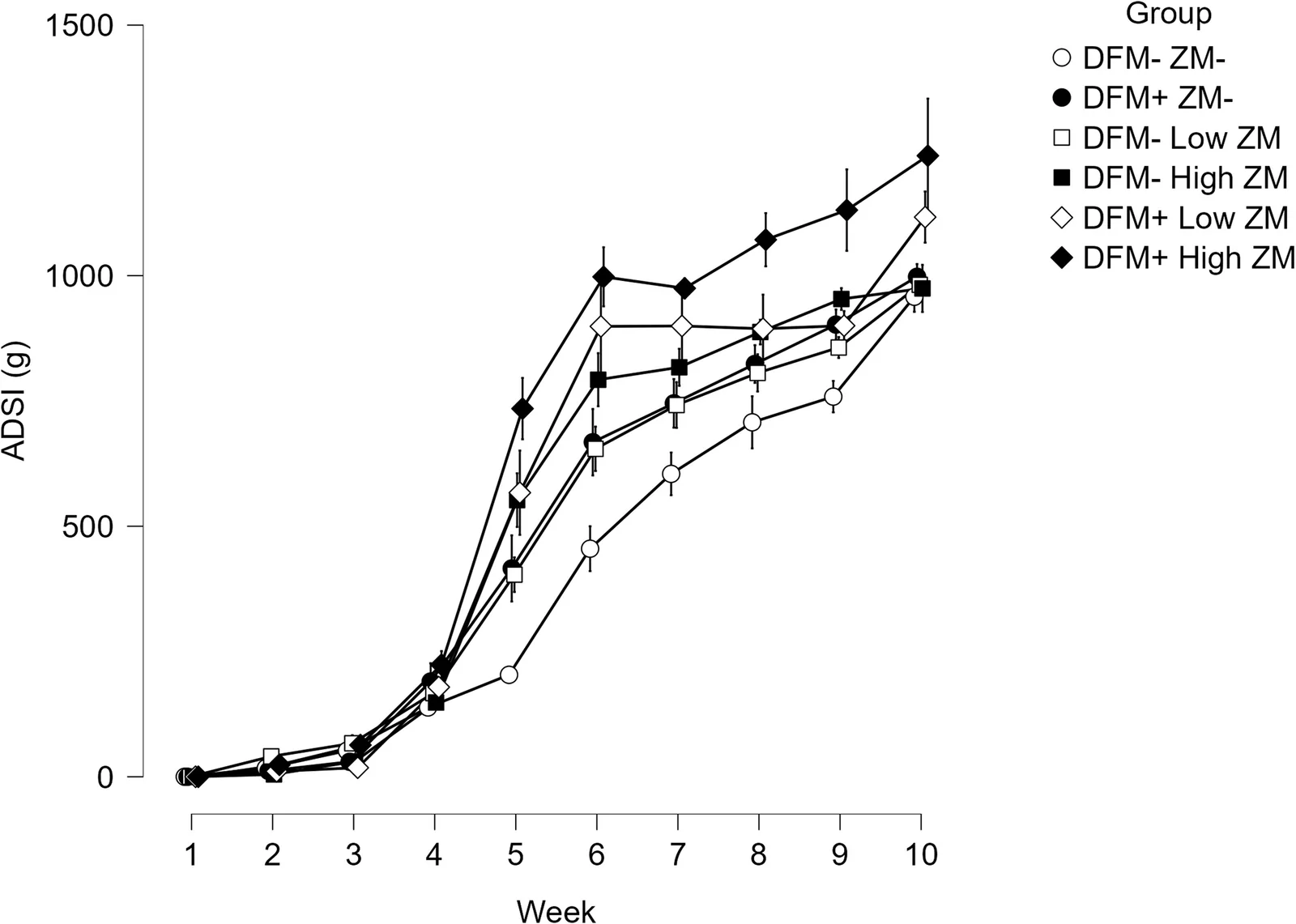
Average daily starter intake (ADSI) in calves receiving whole milk with or without supplementation of direct-fed microbials and 2 levels of zinc-methionine measured by dividing the total weekly starter consumption by number of d in a week. Experimental groups were: (1) No supplementation (DFM- ZM-), (2) Direct-fed microbials supplementation (5 g/calf/d; providing 3 × 10^9^ CFU bacterial strain/calf/d and 5 × 10^8^ CFU yeast/calf/d) without ZM supplementation (DFM+ ZM-), (3) Low ZM supplementation (422 mg/calf/d; providing 80 mg zinc/calf/d) without DFM supplementation (DFM- Low ZM), (4) High ZM supplementation (632 mg/calf/d; providing 120 mg zinc/calf/d) without DFM supplementation (DFM- High ZM), (5) Direct-fed microbials supplementation with low ZM supplementation and (6) Direct-fed microbials supplementation with high ZM supplementation. The error bars represent the standard errors.

Least squares means for BW (Fig. 1), FBW, DMI, ADG, and FCR are presented in Table 4. Initial body weight (IBW) is presented as baseline variable (Table 4). The DFM × ZM interaction numerically increased BW (*P =* 0.08), whereas no interaction was detected for DMI and ADSI. Both DFM and ZM had significant main effects on BW, DMI and ADSI (Fig. 2) (*P <* 0.01). Final body weight (FBW) was not altered by DFM × ZM interaction but it was affected by both DFM (*P =* 0.02), and ZM main effects (*P =* 0.04). While no DFM × ZM interaction was observed for ADG, DFM main effect increased ADG (*P <* 0.01), while the main effect of ZM was not significant. Feed conversion ratio was not affected by DFM × ZM interaction as well as main effect of DFM and ZM. The dose response of ZM was linear for DMI and ADSI (*P <* 0.01), quadratic for FBW (*P =* 0.01), and both linear (*P <* 0.01), and quadratic for BW (*P =* 0.04).

**Table 4:** Least squares mean of performance parameters of preweaning calves fed whole milk with or without supplementation of direct-fed microbials and 2 levels of zinc-methionine.

Item^2^	Supplementation^1^							SEM	*P-*Values										
	DFM-				DFM+				Main effects				ZM Contrast			Interaction effects			
	ZM-	Low ZM	High ZM		ZM-	Low ZM	High ZM		DFM	ZM	Time		Linear	Quadratic		DFM×ZM	DFM×Time	ZM×Time	DFM×ZM×Time
BW (kg)	64.8	66.8	67.9		68.5	67.3	70.6	0.72	<0.01	<0.01	<0.01		<0.01	0.04		0.08	<0.01	<0.01	0.73
IBW (kg)	36.3	38.8	40.0		39.0	37.8	39.5	1.84	0.80	0.51	-		0.58	0.31		0.55	-	-	-
FBW (kg)	88.4	92.3	94.7		95.4	92.7	98.2	1.90	0.02	0.04	-		0.42	0.01		0.23	-	-	-
ADSI (g/d)	389	472	516		478	548	646	23.25	<0.01	<0.01	<0.01		<0.01	0.29		0.49	<0.01	<0.01	0.84
DMI (kg/d)	1.26	1.34	1.38		1.34	1.41	1.49	0.02	<0.01	<0.01	<0.01		<0.01	0.29		0.49	<0.01	<0.01	0.92
ADG (kg/d)	0.744	0.765	0.781		0.805	0.784	0.840	0.02	<0.01	0.11	<0.01		0.12	0.17		0.50	0.14	0.05	0.50
FCR	1.81	2.06	1.89		1.78	1.94	1.99	0.12	0.90	0.20	<0.01		0.15	0.29		0.63	0.50	0.12	0.59

1 DFM = direct-fed microbials supplementation; ZM = zinc-methionine supplementation; DFM- = without direct-fed microbials supplementation; DFM+ = with direct-fed microbials supplementation; ZM- = no zinc-methionine supplementation.

2 BW = body weight; IBW = initial body weight; FBW = final body weight; ADSI = average daily starter intake; DMI = dry matter intake; ADG = average daily gain; FCR = feed conversion ratio. Significance was declared at P *≤* 0.05, and trends at 0.05 *<P≤* 0.10.

### 3.2 Skeletal development

**Fig. 3:**
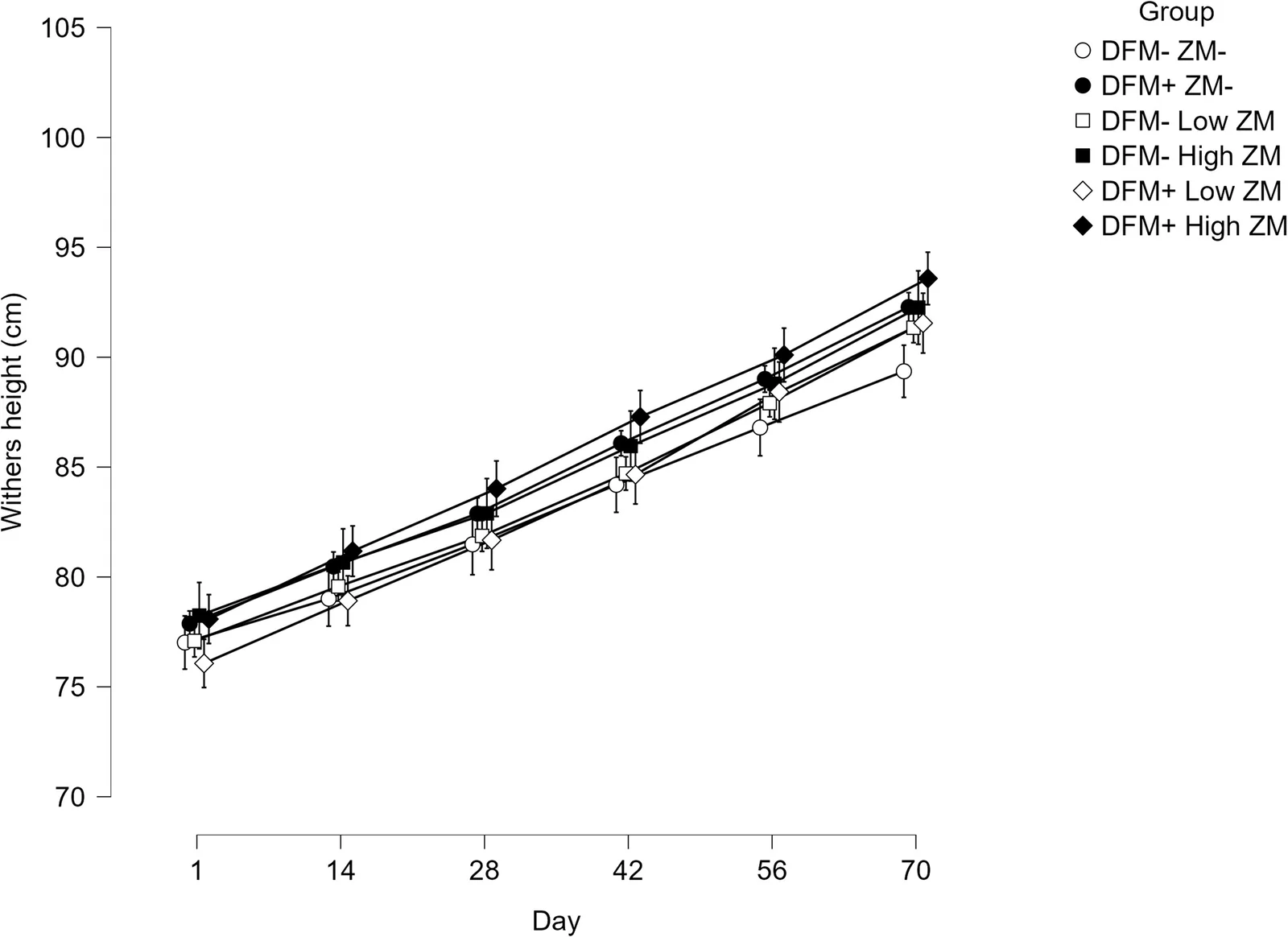
Withers height (WH) in calves receiving whole milk with or without supplementation of direct-fed microbials and 2 levels of zinc-methionine measured on d 1, 14, 28, 42, 56, and 70 of the experiment. Experimental groups were: (1) No supplementation (DFM- ZM-), (2) Direct-fed microbials supplementation (5 g/calf/d; providing 3 × 10^9^ CFU bacterial strain/calf/d and 5 × 10^8^ CFU yeast/calf/d) without ZM supplementation (DFM+ ZM-), (3) Low ZM supplementation (422 mg/calf/d; providing 80 mg zinc/calf/d) without DFM supplementation (DFM- Low ZM), (4) High ZM supplementation (632 mg/calf/d; providing 120 mg zinc/calf/d) without DFM supplementation (DFM- High ZM), (5) Direct-fed microbials supplementation with low ZM supplementation and (6) Direct-fed microbials supplementation with high ZM supplementation. The error bars represent the standard errors.

**Fig. 4:**
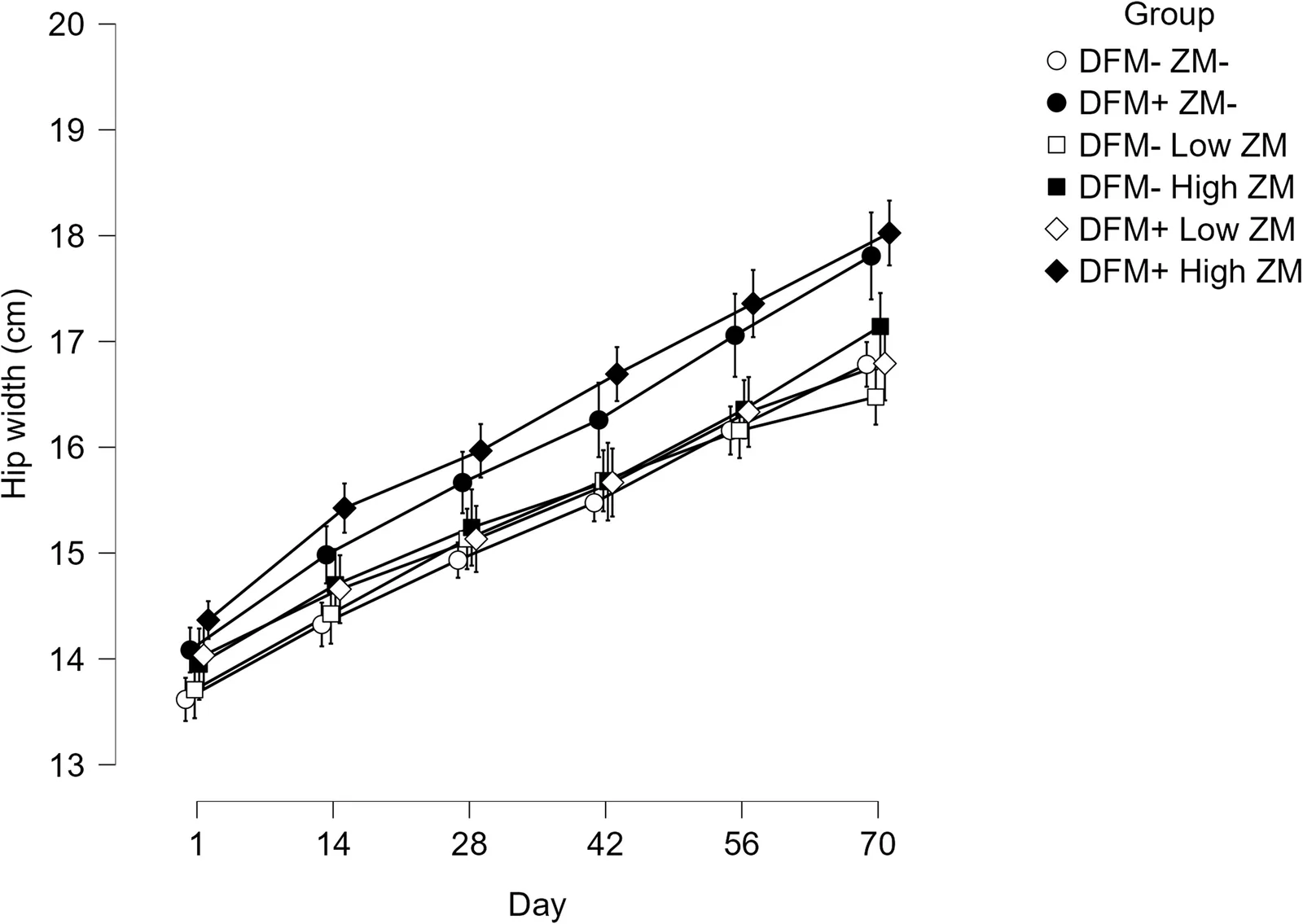
Hip width (HW) in calves receiving whole milk with or without supplementation of direct-fed microbials and 2 levels of zinc-methionine measured on d 1, 14, 28, 42, 56, and 70 of the experiment. Experimental groups were: (1) No supplementation (DFM- ZM-), (2) Direct-fed microbials supplementation (5 g/calf/d; providing 3 × 10^9^ CFU bacterial strain/calf/d and 5 × 10^8^ CFU yeast/calf/d) without ZM supplementation (DFM+ ZM-), (3) Low ZM supplementation (422 mg/calf/d; providing 80 mg zinc/calf/d) without DFM supplementation (DFM- Low ZM), (4) High ZM supplementation (632 mg/calf/d; providing 120 mg zinc/calf/d) without DFM supplementation (DFM- High ZM), (5) Direct-fed microbials supplementation with low ZM supplementation and (6) Direct-fed microbials supplementation with high ZM supplementation. The error bars represent the standard errors.

**Fig. 5:**
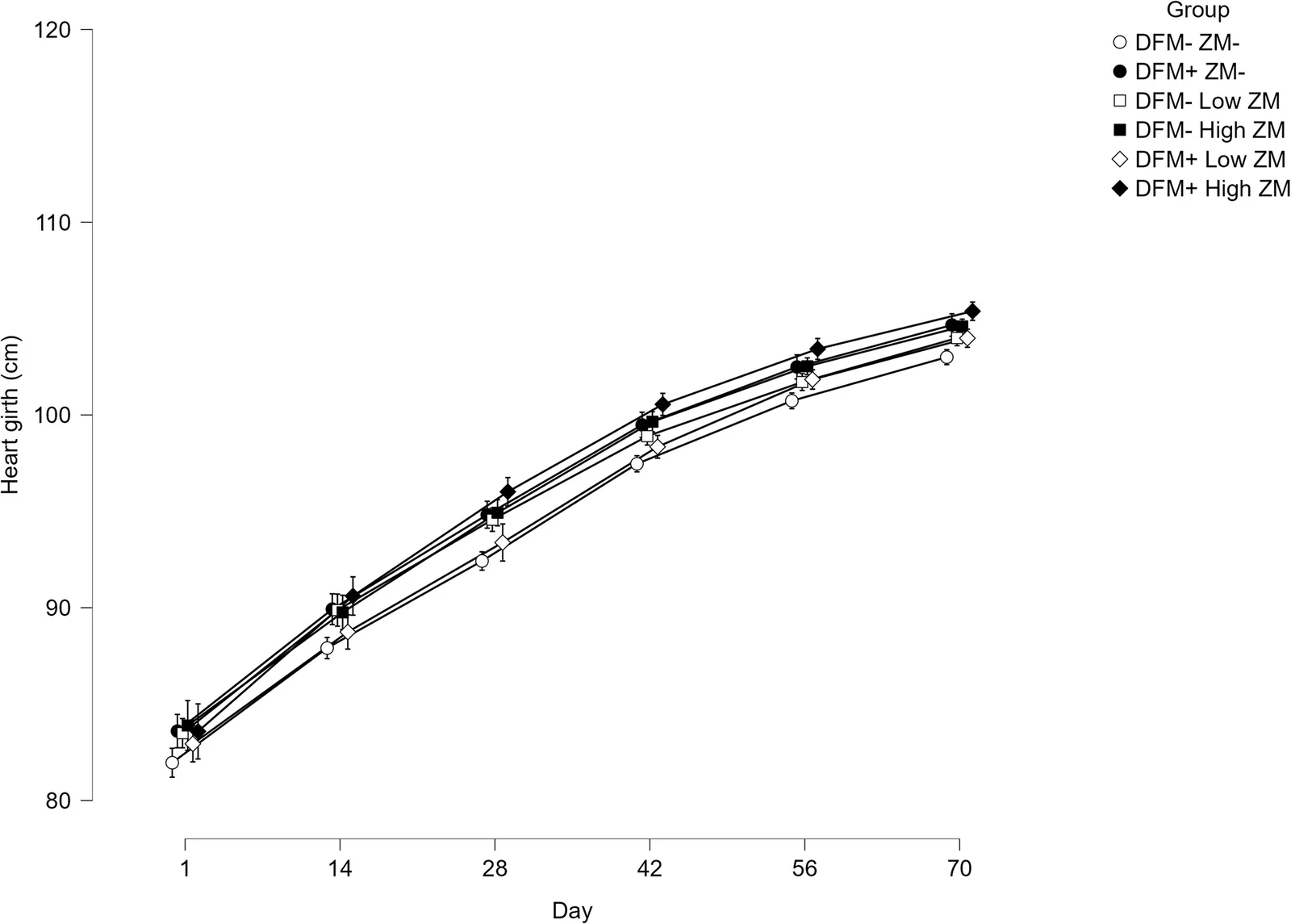
Heart girth (HG) in calves receiving whole milk with or without supplementation of direct-fed microbials and 2 levels of zinc-methionine measured on d 1, 14, 28, 42, 56, and 70 of the experiment. Experimental groups were: (1) No supplementation (DFM- ZM-), (2) Direct-fed microbials supplementation (5 g/calf/d; providing 3 × 10^9^ CFU bacterial strain/calf/d and 5 × 10^8^ CFU yeast/calf/d) without ZM supplementation (DFM+ ZM-), (3) Low ZM supplementation (422 mg/calf/d; providing 80 mg zinc/calf/d) without DFM supplementation (DFM- Low ZM), (4) High ZM supplementation (632 mg/calf/d; providing 120 mg zinc/calf/d) without DFM supplementation (DFM- High ZM), (5) Direct-fed microbials supplementation with low ZM supplementation and (6) Direct-fed microbials supplementation with high ZM supplementation. The error bars represent the standard errors.

Skeletal measurements are reported in Table 5. Withers height (Fig. 3) did not change by DFM × ZM interaction, but it was increased by main effect of DFM (*P <* 0.01) and ZM (*P =* 0.04). For HW (Fig. 4), the DFM × ZM interaction was not statistically significant (*P* = 0.08), but the main effect of ZM was significant (*P =* 0.05), and the estimated HW was numerically greater with DFM supplementation (*P =* 0.07). Heart girth (Fig. 5) was altered significantly with DFM × ZM interaction (*P =* 0.02). The main effects of DFM and ZM also significantly increased HG (*P <* 0.01). Zinc-methionine contrast was linearly significant for WH (*P =* 0.02), quadratically for HW (*P =* 0.01), and both linearly (*P <* 0.01), and quadratically for HG (*P <* 0.01).

**Table 5:** Least squares mean of skeletal development parameters of preweaning calves fed whole milk with or without supplementation of direct-fed microbials and 2 levels of zinc-methionine.

Item^2^	Supplementation^1^							SEM	*P-*Values										
	DFM-				DFM+				Main effects				ZM Contrast			Interaction effects			
	ZM-	Low ZM	High ZM		ZM-	Low ZM	High ZM		DFM	ZM	Time		Linear	Quadratic		DFM×ZM	DFM×Time	ZM×Time	DFM×ZM×Time
WH (cm)	83.3	84.0	83.9		84.3	84.9	85.0	0.30	<0.01	0.04	<0.01		0.02	0.76		0.92	<0.01	0.10	0.49
HW (cm)	15.5	15.5	15.5		15.9	15.4	15.9	0.13	0.07	0.05	<0.01		0.79	0.01		0.08	0.06	<0.01	0. 16
HG (cm)	94.6	95.2	95.5		95.6	95.0	96.4	0.25	<0.01	<0.01	<0.01		<0.01	<0.01		0.02	0.58	0.59	0.62

1 DFM = direct-fed microbials supplementation; ZM = zinc-methionine supplementation; DFM- = without direct-fed microbials supplementation; DFM+ = with direct-fed microbials supplementation; ZM- = no zinc-methionine supplementation.

2 WH = withers height; HW = hip width; HG = heart girth. Significance was declared at *P≤* 0.05, and trends at 0.05 *<P≤* 0.10.

### 3.3 Apparent nutrient digestibility

Apparent digestibility coefficients are shown in Table 6. The DFM × ZM interaction was not significant for DM and OM digestibility. Digestibility of DM (*P =* 0.08) and OM (*P =* 0.09) was numerically greater with DFM supplementation, whereas main effect of ZM was not significant. No DFM × ZM interaction was observed for CP digestibility. Crude protein digestibility was numerically higher in ZM groups (*P =* 0.06), while DFM main effect was not significant. Crude protein digestibility also responded linearly to ZM supplementation (*P =* 0.02). Ether extract and NDF digestibility were not affected by DFM × ZM interaction as well as DFM and ZM main effects.

**Table 6:** Least squares mean of nutrient apparent total tract digestibility of preweaning calves fed whole milk with or without supplementation of direct-fed microbials and 2 levels of zinc-methionine.

Item^2^	Supplementation^1^							SEM	*P-* Values						
	DFM-				DFM+				Main effects			ZM Contrast			Interaction effect
	ZM-	Low ZM	High ZM		ZM-	Low ZM	High ZM		DFM	ZM		Linear	Quadratic		DFM×ZM
DM (%)	70.4	73.6	74.3		78.5	75.4	79.0	3.17	0.08	0.75		0.56	0.62		0.62
OM (%)	71.2	70.5	73.4		72.9	74.8	75.7	1.84	0.09	0.39		0.23	0.54		0.76
CP (%)	70.2	72.7	77.0		71.6	76.1	79.2	2.76	0.32	0.06		0.02	0.61		0.92
EE (%)	79.3	82.1	84.2		83.1	84.9	84.3	2.28	0.24	0.39		0.18	0.89		0.71
NDF (%)	46.0	48.9	47.7		44.8	43.4	46.2	2.21	0.56	0.78		0.50	0.88		0.15

1 DFM = direct-fed microbials supplementation; ZM = zinc-methionine supplementation; DFM- = without direct-fed microbials supplementation; DFM+ = with direct-fed microbials supplementation; ZM- = no zinc-methionine supplementation.

2 DM = dry matter; OM = organic matter; CP = crude protein; EE = ether extract; NDF = neutral detergent fiber. Significance was declared at P ≤ 0.05, and trends at 0.05 < P ≤ 0.10.

### 3.4 Rumen fermentation characteristics

Rumen fermentation variables are presented in Table 7. No effects of DFM × ZM interaction, DFM and ZM main effects, were detected for rumen pH or NH_3__—_N. Total VFA did not alter by DFM × ZM interaction, but it showed higher mean values with DFM main effect (*P =* 0.07), whereas ZM main effect was not significant. Concentrations of individual VFAs were not affected by DFM × ZM interaction, DFM and ZM main effects.

**Table 7:** Least squares mean of ruminal fermentation parameters of preweaning calves fed whole milk with or without supplementation of direct-fed microbials and 2 levels of zinc-methionine.

Item^2^	Supplementation^1^							SEM	*P-* Values^3^						
	DFM-				DFM+				Main effects			ZM Contrast			Interaction effect
	ZM-	Low ZM	High ZM		ZM-	Low ZM	High ZM		DFM	ZM		Linear	Quadratic		DFM×ZM
pH	6.10	6.20	6.30		5.65	5.80	5.60	0.37	0.12	0.94		0.81	0.83		0.91
NH_3—_N (mg/dL)	15.0	13.1	13.9		15.4	14.0	15.0	2.77	0.73	0.84		0.72	0.66		0.99
Total VFA (mM)	102	105	103		108	110	108	2.86	0.07	0.72		0.83	0.45		0.91
Acetate (mM)	54.5	55.3	55.6		53.9	57.5	54.1	2.84	0.98	0.74		0.71	0.51		0.80
Propionate (mM)	32.8	33.7	32.7		36.3	34.9	35.2	2.41	0.27	0.97		0.81	0.94		0.89
Butyrate (mM)	11.2	11.5	11.7		13.9	12.8	13.5	1.24	0.10	0.92		0.97	0.69		0.86
Iso-butyrate (mM)	1.45	1.57	1.30		1.95	1.85	1.90	0.36	0.17	0.94		0.82	0.81		0.90
Valerate (mM)	1.80	2.75	1.95		2.05	2.40	2.25	0.47	0.87	0.42		0.56	0.25		0.76
Iso-valerate (mM)	0.350	0.350	0.300		0.500	0.400	0.550	0.17	0.33	0.95		0.95	0.75		0.85

1 DFM = direct-fed microbials supplementation; ZM = zinc-methionine supplementation; DFM- = without direct-fed microbials supplementation; DFM+ = with direct-fed microbials supplementation; ZM- = no zinc-methionine supplementation.

2 NH_3—_N = ammonia nitrogen; VFA = volatile fatty acid.

3 Significance was declared at P ≤ 0.05, and trends at 0.05 < P ≤ 0.10.

### 3.5 Plasma metabolites

Least squares means for plasma metabolites are summarized in Table 8. No DFM × ZM interaction was detected for any of the plasma metabolites. Plasma glucose increased with main effect of DFM (*P =* 0.03), while ZM main effect was not significant. No effects of DFM main effect or ZM main effect were detected for BHB. Total protein increased with DFM main effect (*P <* 0.01), whereas ZM main effect was not significant. Albumin was affected by the main effect of ZM (*P =* 0.02), whereas no effect of DFM was detected. Urea tended to decrease with the main effect of DFM (*P =* 0.07), with no ZM main effect. Mean plasma creatinine concentration was lower with DFM (*P =* 0.08), while ZM main effect was not significant. Mean plasma triglyceride concentration was higher with DFM supplementation (*P =* 0.07), with no effects of ZM main effect. Cholesterol increased with main effect of DFM (*P =* 0.01), while main effect of ZM was not significant. Plasma albumin level improved linearly by adding ZM supplement (*P =* 0.01).

**Table 8:** Least squares mean of plasma metabolites of preweaning calves fed whole milk with or without supplementation of direct-fed microbials and 2 levels of zinc-methionine.

Item^2^	Supplementation^1^							SEM	*P-*Values										
	DFM-				DFM+				Main effects				ZM Contrast			Interaction effects			
	ZM-	Low ZM	High ZM		ZM-	Low ZM	High ZM		DFM	ZM	Time		Linear	Quadratic		DFM×ZM	DFM×Time	ZM×Time	DFM×ZM×Time
Glucose (mg/dL)	90.9	92.5	98.1		100	101	105	4.22	0.03	0.32	<0.01		0.20	0.42		0.97	0.14	0.40	0.41
BHB (mM)	0.107	0.109	0.112		0.127	0.115	0.144	0.02	0.33	0.80	<0.01		0.73	0.58		0.86	0.20	0.61	0.68
TP (g/dL)	7.63	7.87	7.69		8.08	8.43	8.32	0.16	<0.01	0.22	0.97		0.24	0.20		0.85	0.15	0.12	0.13
Albumin (g/dL)	3.71	3.97	3.96		3.86	4.04	3.99	0.07	0.18	0.02	<0.01		0.01	0.18		0.74	0.82	0.19	0.21
Urea (mg/dL)	12.9	11.1	11.4		11.3	10.6	10.1	0.73	0.07	0.14	<0.01		0.06	0.57		0.75	0.87	0.60	0.45
Creatinine (mg/dL)	1.41	1.17	1.14		0.979	1.03	1.02	0.16	0.08	0.73	0.06		0.44	0.88		0.54	0.41	0.79	0.55
Triglyceride (mg/dL)	30.2	34.1	34.5		39.5	43.1	47.4	6.58	0.07	0.65	<0.01		0.36	0.95		0.95	0.22	0.81	0.14
Cholesterol (mg/dL)	84.9	88.4	90.2		103	96.7	111	6.78	0.01	0.48	<0.01		0.46	0.34		0.63	0.69	0.41	0.52

1 DFM = direct-fed microbials supplementation; ZM = zinc-methionine supplementation; DFM- = without direct-fed microbials supplementation; DFM+ = with direct-fed microbials supplementation; ZM- = no zinc-methionine supplementation.

2 BHB = β-hydroxybutyrate; TP = total protein. Significance was declared at *P≤* 0.05, and trends at 0.05 *<P≤* 0.10.

## 4 Discussion

In the present study, lack of improvement in FCR suggests that higher intake might be the main reason of improvement in body mass and frame development by DFM and ZM supplementation (Table 4). This interpretation is consistent with recent meta-analysis on DFM and zinc supplementation in preweaning calves (Branco-Lopes et al., 2025; Hashemi, 2025).

Because no significant DFM × ZM interactions were detected except HG, the responses for the remaining traits are discussed primarily in terms of the main effects of DFM and ZM (Cavalcanti et al., 2010). In contrast, interpretation of HG was based on the significant DFM × ZM interaction, and discussion of the corresponding main effects was intentionally avoided because the treatments are responding differently within a main effect (Cavalcanti et al., 2010).

Consistent with our study, Liu et al. (2023a) reported that organic zinc supplementation significantly increased intake in preweaning calves. However, our results differ from those of Rajaei-Sharifabadi et al. (2024) who found no significant effect of organic zinc supplementation on feed intake in preweaning calves. Duration of zinc supplementation in both studies was shorter than our study, so the discrepancy with the findings of Rajaei-Sharifabadi et al. (2024) is more likely related to environmental conditions. Compared with our study, their trial was conducted under higher average ambient temperature (38 °C vs 26 °C) while Liu et al. (2023a) conducted their study during autumn. Pro-inflammatory cytokines are one of the most documented factors that may reduce appetite in ruminants under stress, such as transition cows and preweaning calves (Brown & Bradford, 2021). Zinc may mitigate the effects of chronic inflammation on appetite by inhibiting the transcription factor NF-κB which is necessary for pro-inflammatory gene expression, such as IL-6 and TNF-α (Chang et al., 2024). This mechanism aligns with Chen et al. (2020) where DMI linearly increased, while plasma IL-6 linearly decreased by increasing ZM supplementation in prepartum dairy cows. Albumin is one of the negative acute phase protein that decreases in ruminants with inflammatory condition (Tothova et al., 2014). In the present study, Albumin increased linearly as ZM supplementation increased (Table 8).

Some previous studies have reported that DFM effectively improved starter intake in preweaning dairy calves which aligns with the present study (Ayyat et al., 2023; Jiang et al., 2020). Davies et al. (2025) also demonstrated that *Saccharomyces cerevisiae* supplementation increased starter intake in preweaning calves under chronic stress. However, some other studies have indicated that starter intake did not change in preweaning calves fed DFM (Guo et al., 2021; Ma et al., 2025). The variation among studies might be related to duration or dose of the supplementation. For instance, both Ayyat et al. (2023) and Jiang et al. (2020) used a high dose of DFM supplements from first week of age until weaning which was similar to the present study. In contrast, Guo et al. (2021) used a lower dose of DFM only for one month. In addition, the study by Ma et al. (2025) not only had a shorter duration compared to the present study but the start of supplementation was also different (4 vs 35 d of age). In our study, the improvement of intake by DFM might be related to the numerical improvements in DM and OM digestibility in DFM groups (Table 6). In ruminants, greater digestibility supports intake by increasing the digesta clearance (NASEM, 2021). This interpretation is consistent with Rahimi et al. (2023) who observed more intake in preweaning calves with improved DM and OM digestibility.

In one study conducted on 11 commercial dairy farms, 70% of preweaning calves grew below the recommended 0.7 kg/d, indicating that even relatively small improvements in daily gain may be operationally meaningful under commercial conditions (Johnson et al., 2017). In the present study, a 47 g/day increase in ADG (Table 4) would accumulate to approximately 3.3 kg over a 70-day preweaning period, which is modest but still relevant when calves are managed close to growth targets. This response is likely to be economically meaningful if it persists beyond weaning, because preweaning ADG has been positively associated with first-lactation milk yield, and a meta-analysis reported that maintaining ADG above 0.5 kg/day can enhance first lactation performance (Gelsinger et al., 2016). Furthermore, improved early growth is important because delayed age at first calving increases rearing costs (Wathes et al., 2014). In accordance, Boulton et al. (2017) stated that each extra day of age at first calving increased mean rearing cost by £2.87.

Khademi et al. (2022) demonstrated that DFM supplementation improved HW in preweaning calves, in the present study also the estimated HW was numerically greater in DFM group. Moreover, Yadav et al. (2024) reported improvements in WH during the preweaning period by DFM supplementation. In most previous studies, ZM supplementation has not been reported to improve skeletal dimensions (Ma et al., 2020; Rajaei-Sharifabadi et al., 2024; Wo et al., 2022). However, in these studies ZM has been supplemented only for two weeks which might be too short to expect differences in frame, while in this study ZM was supplemented for ten weeks. This interpretation aligns with Schwarzkopf et al. (2019) who observed significant improvements in frame after 56, 84, 126 and 140 d due to nutritional and management interventions in preweaning Holstein calves.

In this study, the DFM × ZM interaction was observed only for HG, whereas the other growth traits showed additive responses or no interaction (Table 5). In a large study including 6306 dairy cows, HG yielded the lowest root mean square error (RMSE) for body weight prediction compared with other linear body measurements, indicating that it captured variation in body size more precisely (Gruber et al., 2018). In addition, Heinrichs et al. (2007) demonstrated that HG measurements are highly repeatable, with repeatability exceeding 0.99 both within and between observers, indicating minimal measurement error and excellent measurement consistency under field conditions. Consequently, if the DFM × ZM interaction was relatively small, it would be statistically more likely to be detected in heart girth than in traits with lower measurement precision or weaker association with overall body size. The observed DFM × ZM interaction detected for a single outcome should be regarded as a trait-specific finding rather than evidence of a consistent biological interaction across all measured variables (Izmirlian et al., 2025). Therefore, although the interaction observed for HG may represent a genuine response, its interpretation should remain confined to this specific trait until confirmed in independent studies.

In the present study, significant DFM × Time and ZM × Time interactions were detected for several performance variables including BW, ADG, DMI, ADSI, WH and HW (Table 4, Table 5). This temporal pattern is biologically plausible because preweaning calves undergo rapid gastrointestinal, metabolic, and immune maturation during the first weeks of life, which can progressively modify their responsiveness to nutritional interventions (Du et al., 2023; Fischer et al., 2019; Malmuthuge et al., 2015). This is in line with Biricik et al. (2023), who observed significant treatment × week interactions for BW, ADG, and feed intake, with treatment differences becoming evident only during the later stages of the preweaning period. Likewise, Liu et al. (2023b) reported significant treatment × day interactions for DMI and FCR following zinc proteinate supplementation.

In this study, DFM supplementation resulted in numerically greater apparent digestibility of DM (*P =* 0.08), and OM (*P =* 0.09), together with higher mean total VFA concentration in rumen fluid (*P =* 0.07). There is lack of evidence about efficacy of DFM on digestive and rumen parameters throughout the literature but it has been investigated only in a few studies (Wang et al., 2023). In Khademi et al. (2022) supplementation of DFM did not affect total VFA concentration in rumen fluid or DM and OM digestibility. The DFM used in their study was also a mixed product with almost the same bacterial and yeast strains used in this study, but the daily supply of bacterial strains and yeast was lower than the present study (2 × 10^8^ CFU/calf vs 3 × 10^9^ CFU/calf) (Khademi et al., 2022). Wang et al. (2022) reported that the effect of a compound DFM containing *Pediococcus, Bacillus* and *Lactobacillus* spp on total VFA was not significant. The daily amount of DFM supplementation in this study was even lower than Khademi et al. (2022) (1.2 × 10^7^ and 1.2 × 10^8^ vs 2 × 10^8^ CFU/calf). Moreover, in these studies DFM did not change starter intake which is inconsistent with the present study (Khademi et al., 2022; Wang et al., 2022). Accordingly, it has been shown that higher intake improves total VFA in rumen fluid by increasing the amount of fermentable organic matter in preweaning calves (Beiranvand et al., 2019; Yazdanyar et al., 2025).

In the present study CP digestibility showed higher mean values in ZM group (*P =* 0.06) and increased linearly as the supplementation level increased (*P* = 0.02). This pattern is consistent with improvement plasma albumin in the present study (Table 8) as well as intake and growth responses (Table 4). However, ZM did not alter plasma TP and urea (Table 8), or ruminal NH_3__—_N (Table 7). In ruminants ruminal NH_3__—_N and plasma urea are indicators of protein degradation in rumen (Broderick & Clayton, 1997). So, the observed pattern may reflect enhanced intestinal enzyme activity in response to ZM supplementation. Accordingly, zinc dependent peptidases have been reported to have a key role in protein and peptide digestion in small intestine (Cai et al., 2025). Albumin has a faster turnover rate than other serum proteins, making it a more reliable indicator of protein availability (Siachos et al., 2024), while total protein in addition to albumin contains other proteins such as globulins which often remain stable or increase during inflammation which can mask variations in albumin within the TP measure (Duvall et al., 2023). This is consistent with several studies in which higher protein supply changed serum albumin but not TP in calves (Chen et al., 2023; Manso et al., 2025).

According to Table 8, the main effect of DFM significantly increased plasma glucose, cholesterol and TP. Direct-fed microbials were also associated with higher mean plasma triglyceride concentrations and lower mean plasma urea and creatinine concentrations. In the present study, the increase in plasma glucose in DFM groups may reflect greater systemic nutrient availability, which is consistent with the higher starter intake (Table 4), together with higher mean digestibility coefficients (Table 6) and total ruminal VFA concentration (Table 7). Jiang et al. (2020) also reported the same pattern in which supplementation of *Lactobacillus plantarum* increases intake and subsequently plasma glucose level in preweaning calves.

The plasma TP mainly includes albumin and globulins (mainly IgG, IgA and IgM) (Duvall et al., 2023). In our experiment the plasma albumin did not change by DFM supplementation (Table 8) so the improvement in TP is likely more related to globulins. Despite the lack of globulin data particularly immunoglobulins in the present study, several studies have indicated that DFM supplementation could increase serum immunoglobulins in preweaning dairy calves (Ayyat et al., 2023; Cai et al., 2024; Lu et al., 2022).

In the present study, the apparent digestibility of EE did not change by DFM (Table 6) while DMI intake increased significantly (Table 4). Therefore, the higher plasma cholesterol concentration together with the higher mean plasma triglyceride concentration may reflect greater nutrient flux rather than increased lipid digestibility.

Higher concentration of plasma urea is an indicator of protein utilization in ruminants (Hristov et al., 2019). So, the lower mean plasma urea concentration observed with DFM supplementation may be compatible with more efficient nitrogen utilization despite the higher feed intake. Moreover, in a recent study by Kessler et al. (2024) in dairy cows it has been noted that high circulating urea and creatinine are related to kidney dysfunction. In this study, lower mean plasma urea and creatinine concentrations in DFM groups do not suggest an increased metabolic burden on the kidneys.

In the present study zinc concentrations in the diet were not analytically determined, instead, dietary zinc values were estimated using NASEM-Dairy-8 software. So, the present findings should be interpreted as responses to supplementation level rather than as responses to dietary zinc. Future studies using designs to evaluate zinc at nutrient level would help to optimize zinc requirements in preweaning calves while preserving the practical supplementation strategies. Additionally, serum Immunoglobulins, pro-inflammatory cytokines and microbiome data would help the interpretation of performance data for both DFM and zinc supplementation. In addition, the viability of the DFM product was not independently verified throughout the experimental period; therefore, the stability of viable microorganisms under the study conditions could not be confirmed. Although all calves received colostrum according to the farm's standard management protocol immediately after birth, variation in passive immunity among calves cannot be completely excluded. Therefore, a potential influence of passive transfer status on the measured responses should be considered when interpreting the findings. Another limitation of this study is that a formal a priori sample size calculation was not performed because the number of eligible calves was constrained by farm availability and practical considerations. In addition, because multiple prespecified outcomes were evaluated, the possibility of Type I error inflation due to multiple comparisons should be considered when interpreting the findings. Finally, future studies should account for the methionine contribution of ZM during diet formulation and monitor plasma methionine status to better distinguish the effects of zinc and methionine.

## 5 Conclusions

Overall, supplementing DFM or ZM was associated with better growth performance in preweaning dairy calves. Also, higher level of ZM showed a relative superiority compared to lower level to enhance performance. But our results suggest that the improvements were not accompanied by better FCR, indicating that the main response was driven by higher starter intake. Moreover, according to our study there might not be any notable interaction between DFM and ZM in order to improve growth performance in preweaning dairy calves except for HG.

**Abbreviations**

**Table:** 

**ADSI**	average daily starter intake	**DM**	dry matter
**BHB**	β-hydroxybutyrate	**DMI**	dry matter intake
**FBW**	final body weight	**EE**	ether extract
**DFM**	direct-fed microbials	**FCR**	feed conversion ratio
**HG**	heart girth	**ME**	metabolizable energy
**HW**	hip width	**NDF**	neutral detergent fiber
**IBW**	initial body weight	**NH3-N**	ammonia nitrogen
**TP**	total protein	**OM**	organic matter
**WH**	withers height	**VFA**	volatile fatty acids
**ZM**	zinc-methionine	**ADG**	average daily gain
**BW**	body weight		
**CP**	crude protein		

## Ethics declaration

This study was conducted in accordance with the ARRIVE (Animal Research: Reporting of In Vivo Experiments) guidelines. This study was approved by the Animal Ethics Committee of College of Agriculture and Natural Resources, University of Tehran. (Approval No. AEC-202504-1011) Statement for studies in animals The present study was authorized by the Animal Ethics Committee of College of Agriculture and Natural Resources, University of Tehran, with the authorization number AEC-202504-1011.

## CRediT authorship contribution statement

**Ahmadreza Alipour:** Writing – review & editing, Writing – original draft, Visualization, Methodology, Investigation, Formal analysis, Data curation, Conceptualization. **Mahdi Ganjkhanlou:** Writing – review & editing, Validation, Supervision, Resources, Project administration, Methodology, Funding acquisition. **Abolfazl Zali:** Writing – review & editing, Validation, Supervision, Methodology. **Mahdi Dehghan-Banadaki:** Writing – review & editing, Resources, Methodology. **Hossein Esmaeili:** Writing – review & editing, Methodology. **Mohammad Ramin:** Writing – review & editing, Visualization, Validation, Supervision, Data curation.

## Declaration of competing interest

The authors declare that they have no known competing financial interests or personal relationships that could have appeared to influence the work reported in this paper.

## Data Availability

The data that support the results of the present study are available from the corresponding authors upon reasonable request.
